# Fused IgY Fc and Polysaccharide Adjuvant Enhanced the Immune Effect of the Recombinant VP2 and VP5 Subunits—A Prospect for Improvement of Infectious Bursal Disease Virus Subunit Vaccine

**DOI:** 10.3389/fmicb.2017.02258

**Published:** 2017-11-14

**Authors:** Huining Wang, Sufeng Shan, Shujuan Wang, Hao Zhang, Lili Ma, Liping Hu, He Huang, Kai Wei, Ruiliang Zhu

**Affiliations:** ^1^Shandong Provincial Key Laboratory of Animal Biotechnology and Disease Control and Prevention, Shandong Agricultural University, Tai'an, China; ^2^Shandong Provincial Engineering Technology Research Center of Animal Disease Control and Prevention, Shandong Agricultural University, Tai'an, China; ^3^Animal Disease Prevention and Control Center of Shandong Province, Animal Husbandry and Veterinary Bureau of Shandong Province, Jinan, China; ^4^New Hope Group, Shandong New Hope Liuhe Co. Ltd., Qingdao, China

**Keywords:** IBDV, IgY Fc, *Pichia pastoris* expression, subunit vaccine, TPPPS

## Abstract

Infectious bursal disease virus (IBDV) is a highly contagious pathogen that causes damage in lymphoid organs and remains a threat to the poultry industry worldwide. Currently, subunit vaccines based on VP2 antigen expressed in prokaryotic systems are widely used in clinical settings. However, the immunogenicity of VP2 vaccines is limited because of their inherent defect that the structure of the antigen expressed in *Escherichia coli* (*E. coli*) may be different from its natural conformation. In this study, we fused VP2 and VP5 protective antigen genes and linked the chicken IgY Fc gene onto it. The eukaryotic expression plasmid carrying the fusion gene was transformed into *Pichia pastoris* (*P. pastoris*) to express the recombinant VP2–VP5–Fc protein. The recombinant protein was used as immunogen for evaluating immune response, and the recombinant VP2–Fc and VP2 proteins expressed in *P. pastoris* and the commercial VP2 subunit vaccines were used as controls. Moreover, Taishan *Pinus massoniana* pollen polysaccharide (TPPPS), an immunomodulator found by our laboratory, was used as adjuvant to investigate its immune modulatory effects on immunogens. Chickens were divided into six groups and inoculated with VP2–VP5–Fc+TPPPS, VP2–VP5–Fc, VP2–Fc, VP2 vaccine, commercial VP2 subunit vaccine, and phosphate buffered saline (PBS). The recombinant VP2 subunit vaccine expressed in *P. pastoris* exhibited higher immunogenicity than the commercial VP2 subunit vaccine. The VP2–Fc protein showed a better effect than the VP2 protein, and the VP2–VP5–Fc subunit further improved the immune effects. In addition, TPPPS was proved to be a good immunopotentiator for the VP2–VP5–Fc subunit vaccine. Hence, the recombinant VP2–VP5–Fc subunit combined with TPPPS adjuvant exhibits potential as efficient IBDV vaccine to prevent infectious bursal disease.

## Introduction

Infectious bursal disease (IBD) is a highly contagious and immunosuppressive disease in young chickens and still poses a potential threat to the poultry industry now. IBD is caused by the IBD virus (IBDV), a non-enveloped RNA virus in the family of *Birnaviridae* (Mundt et al., 1995), which mainly destroys the B lymphocyte precursors in bursa that leads to the lymphoid depletion of B cells and marked atrophy of the bursa, thereby leading to the severe immunosuppressive disease (Sharma et al., 2000; Yao and Vakharia, 2001; Liu and Vakharia, 2004). Currently, besides the conventional live attenuated and inactivated vaccines, IBDV VP2-based subunit vaccine expressed in *Escherichia coli* (*E. coli*) has been extensively used in clinical settings (Pradhan et al., 2012). *E. coli* expression system has been recognized as the most convenient, economical, and fastest expression system owing to the ease of growth and genetic manipulation (Terpe, 2006; Chen, 2012). However, the lack of appropriate post-translational processing mechanisms in *E. coli* leads to the great difference between the expressed viral epitopes and its natural structure (Gonzalez-Montalban et al., 2007; Martinez-Alonso et al., 2008). Thus, the immunogenicity of the VP2 subunit vaccine is also limited. How to enhance the immunogenicity of IBDV subunit vaccine is crucial for preventing this disease.

VP2 protein, encoded by a 1,362 bp gene fragment, is the major host-protective antigen of IBDV, which contains the major neutralization sites. The truncated fragment (VP2_52−417_), which includes key neutralization epitopes, has been identified to possess potent antigenicity (Pradhan et al., 2012). In addition, VP5 protein, a 17-kDa nonstructural polypeptide, is inessential for virus replication *in vitro* but bears important responsibility for pathogenesis and dissemination (Mundt et al., 1997). This protein accumulates with the host plasma membrane and triggers the release of viral particles (Lombardo et al., 2000). VP5 protein also has effective immunogenicity and induces the production of high titer polyclonal antibodies (Zhang et al., 2007). Hence, we pose to obtain the recombinant subunits of VP2 and VP5 proteins via fusion expression techniques to improve the immunogenicity of subunit vaccines.

Mammalian IgG molecule can be divided into two Fab regions for binding to highly variable antigens and an Fc portion for recruiting and activating immune effector leukocytes. The engagement of Fc with activating Fc receptor (FcR) increases the efficiency of these antigen-presenting cells for antigen elimination and triggers effector functions for the removal of defective cells (Jefferis, 2009a,b; Schwab and Nimmerjahn, 2013). Moreover, the antigen-antibody complex can avoid degradation in lysosomes by interacting with FcR, resulting in its prolonged half-life *in vivo* (Kuo et al., 2010). In avian species, although the Fc segments between IgG and IgY demonstrate different structures, IgY is similar to mammalian IgG in terms of functionality (Linden and Roth, 1978; Tressler and Roth, 1987). Our previous studies have found that chicken IgY Fc portion stimulates the activation of macrophages and promotes the antigen-processing efficiency, thereby enhancing the immune response induced by the antigen (Dong et al., 2016). Therefore, the introduction of chicken IgY Fc is potential to improve immune effects of IBDV subunit vaccines.

Considering the factors that may affect the immune efficacy of IBDV subunit vaccines, we designed and constructed a recombinant plasmid, which expresses the VP2 and VP5 of IBDV and the chicken IgY Fc fusion protein. *Pichia pastoris* (*P. pastoris*) eukaryotic expression system, which is capable of expressing proteins with their correct folding and several posttranslational modifications, was employed as a well-established host for the expression of the recombinant protein. Moreover, Taishan *Pinus massoniana* pollen polysaccharides (TPPPS) has been proved to be an effective adjuvant for improving the immune system and facilitating immune responses in our laboratory (Wei et al., 2011; Cui et al., 2013; Wang et al., 2013). Herein, TPPPS was used as adjuvant to investigate its immune modulatory effects on the recombinant subunit antigen. Finally, this several-fold improved immunogen was used to evaluate its immune effect in chickens.

## Materials and methods

### Ethics statement

The animal procedures performed were reviewed and approved by the Animal Care and Use Committee of Shandong Agricultural University (permit number: 20010510) and performed in accordance with the “Guidelines for Experimental Animals” of the Ministry of Science and Technology (Beijing, China).

### Strains, plasmids, and vaccines

The vv IBDV GX8/99 strain preserved in our laboratory was isolated from Guangxi and has been adapted in chicken embryo fibroblast cells. *P. pastoris* GS115 and plasmid pPIC9 were purchased from Invitrogen (Carlsbad, CA, USA). Commercial VP2 subunit vaccine was acquired from Qingdao Yebio Biological Engineering Co. Ltd. All media were prepared according to the manuals of *Pichia* expression.

### Construction of recombinant expression vector

Polymerase chain reaction (PCR) was used to amplify the genes. The VP2 fragments of VP2, VP2-Fc, and VP2-VP5-Fc genes were amplified with three pairs of primers (VP2-F_1_ and VP2-R_1_; VP2-F_1_ and VP2-R_2_; VP2-F_1_ and VP2-R_3_), respectively; VP5 gene was amplified with a pair of primers (VP5-F_1_ and VP5-R_1_); the IgY Fc fragments of VP2-Fc and VP2-VP5-Fc fusion genes were amplified with two pairs of primers (Fc-F_1_ and Fc-R_1_; Fc-F_2_ and Fc-R_1_), respectively. Then, VP2–Fc fusion gene was amplified with a pair of primers (VP2-F_1_ and Fc-R_1_) by overlapping PCR, VP2–VP5 fragment of VP2–VP5–Fc fusion gene was amplified with a pair of primers (VP2-F_1_ and VP5-R_1_), and VP2–VP5–Fc fusion gene was amplified with a pair of primers (VP2-F_1_ and Fc-R_1_) by overlapping PCR. The primers were designed according to the VP2 and VP5 gene sequences of IBDV (GenBank accession number: AY907014.1 and EF101893.1) and chicken IgY Fc gene (GenBank accession number: X07174). All primers were designed using Primer 5.0 software, and these primers used for construction of these genes were listed in Table 1. The fused genes were cloned into the pPIC9 vector and confirmed by sequencing (TSINGKE, Beijing), namely, pPIC9-VP2, pPIC9-VP2–Fc, and pPIC9-VP2–VP5–Fc. The recombinant plasmids were transformed into *E. coli* DH5α strain to expand the yield.

**Table 1:** The primers used in this study.

**Primer name**	**Sequence(5′-3′)^A^**
VP2–F_1_	CC*CTCGA*GATGGCAGCCGATGATTACCAATTCT
VP2–R_1_	TTGCGGCCGCTTAATGATGATGATGATGATGGAGGTCGGCCACCTCCATGAAG
VP2–R_2_	CGTATGAACCTCCACCTCCTGATCCACCTCCACCGAGGTCGGCCACCTCCAT
VP2–R_3_	ATAGTGAACCTCCACCTCCTGATCCACCTCCACCGAGGTCGGCCACCTCCAT
VP5–F_1_	CCTCGGTGGAGGTGGATCAGGAGGTGGAGGTTCACTATCATTGATGGTTAG
VP5–R_1_	CGTATGAACCTCCACCTCCTGATCCACCTCCACCCTCAGGCTTCCTTGGAAG
Fc–F_1_	CCTCGGTGGAGGTGGATCAGGAGGTGGAGGTTCATACGCCATCCCACCCA
Fc–R_1_	TT*GCGGCCGC*TTAATGATGATGATGATGATGGCGCTGGCTGAAGCGGATG
Fc–F_2_	TGAGGGTGGAGGTGGATCAGGAGGTGGAGGTTCATACGCCATCCCACCCAGC

A *The italic bases encode XhoI (CTCGAG) and NotI (GCGGCCGC) restriction sites, and the underlined bases encode flexible linker peptides*.

### Expression, purification, and identification of the recombinant proteins

The constructed recombinant plasmids were transformed into competent *P. pastoris* GS115 strain to obtain the pPIC9-VP2, pPIC9-VP2–Fc, and pPIC9-VP2–VP5–Fc *P. pastoris* transformants. *P. Pastoris* transformed with blank pPIC9 plasmid served as the negative control. The protein expression was induced by methanol. The culture supernatants were harvested through centrifugation at 24, 48, 72, and 96 h post methanol induction. The recombinant proteins were purified through ProteinIso™ Ni-NTA Resin kit (TRANS, Beijing, China) and identified by SDS-PAGE and Western blot analysis as described in a previous study (Temple et al., 2010). Protein concentration was determined by Easy II Protein Quantitative Kit (BCA) (TRANS, Beijing, China).

### Preparation of vaccine

TPPPS was prepared through hot water extraction and ethanol precipitation as described previously in our laboratory (Wei et al., 2011). The purified recombinant VP2–VP5–Fc protein was mixed with TPPPS at a ratio of 1:1, reaching a final concentration of 10 mg/mL, with the TPPPS at doses of 50 mg/mL. Then the stability and sterility tests were performed to evaluate the recombinant subunit vaccines.

### Animal experiment

A total of 300 1-day-old male specific pathogen-free (SPF) white leghorn chickens (Spirax Ferrer Poultry Co. Ltd., Jinan) were randomly divided into six sterilized isolators on average (namely, groups I–VI). The ambient conditions were set at 20–25°C and 30–40% relative humidity, and the air entering the isolators was filtered. All chickens in groups I–VI were subcutaneously inoculated with 0.2 mL of TPPPS adjuvant VP2–VP5–Fc subunit vaccine, pure VP2–VP5–Fc subunit vaccine, VP2–Fc subunit vaccine, VP2 subunit vaccine, commercial VP2 vaccine, and phosphate buffered saline (PBS) at 7 days old. At 0, 7, 14, 21, 28, 35, 42, and 49 days post vaccination (dpv), three chickens from each group were selected randomly to evaluate the relevant immune indexes. The chickens were starved for 12 h before sampling.

Three weeks after the vaccination (21 dpv), 30 chickens from each group were placed in a new isolator and challenged intranasally with 2,000 median embryo lethal dose (ELD_50_) of the virulent IBDV. Clinical symptoms and survival status of the chickens were monitored for 7 days after challenge. Bursal lesion scores were evaluated with the corresponding methods. The protection and morbidity rates in each group were calculated according to the following formulas:

$$\begin{array}{l} \text{Protective rate}\left(\%\right)=\text{No}.\text{ of chickens without clinical}\\ \text{symptoms}/\text{Total No}.\;\times 100\%.\\ \text{Morbidity}\left(\%\right)=\text{No}.\text{ of chickens with clinical symptoms}/\\ \text{Total No}.\;\times 100\%. \end{array}$$

### Detection of serum antibody titers and cytokine concentration

Three serum samples from each group were randomly collected at the sampling time. An indirect enzyme-linked immunosorbent assay (ELISA) was developed to detect the antibody titers according to a previously reported method (Denac et al., 1997). The cytokines of IL-2, IL-4, and IFN-γ were detected by the chicken IL-2, IL-4, and IFN-γ ELISA kits (Langdon Bio-technology Co. Ltd., Shanghai). The absorbance was determined at 450 nm in a microplate reader.

### Peripheral blood lymphocyte proliferation

Lymphocyte proliferation was assessed by MTT method. In brief, fresh anticoagulated peripheral blood samples from three chickens (1.0 mL/chicken) in each group were randomly collected and mixed with 1.0 mL of PBS separately. Then, 2 ml of mixture was added to 5 ml of lymphocyte separation medium (Solarbio, China) to separate lymphocytes (Mwanza et al., 2009). Then the lymphocyte proliferation assay was performed as previously described (Mosmann, 1983).

### Detection of CD4^+^ and CD8^+^ T lymphocyte counts in peripheral blood

Fresh anticoagulated peripheral blood samples from three chickens (1.0 mL/chicken) in each group were randomly collected and mixed with 1.0 mL of PBS separately. Then, 2 ml of mixture was added to 5 ml of lymphocyte separation medium (Solarbio, China) to separate lymphocytes (Mwanza et al., 2009). The percentages of CD4^+^ and CD8^+^ T lymphocytes were detected by flow cytometry (Guaga Easy Cyte Mini, USA).

### Statistical analysis

Data were expressed as mean ± *SD*, and Duncan's multiple-range test was performed to analyze the differences among groups using SPSS 17.0. *P* < 0.05 was considered statistically significant.

## Results

### Construction, expression, and identification of recombinant proteins

**Figure 1:**
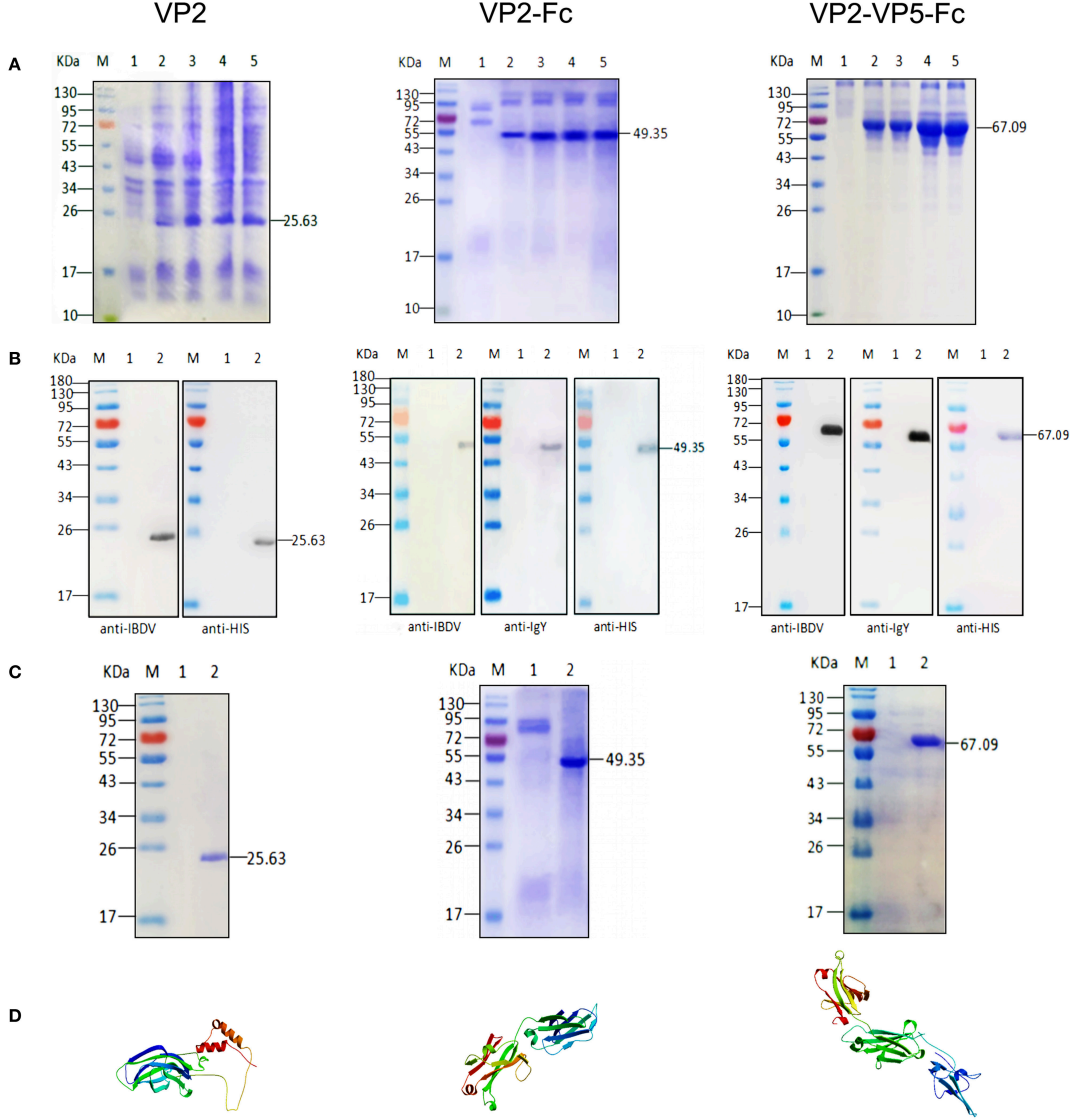
Expression, SDS–PAGE identification, and Western blot analysis of the recombinant proteins. **(A)** SDS–PAGE identification of the recombinant VP2, VP2–Fc, VP2–VP5–Fc at different induction times. M, Page ruler pre–stained protein ladder; lane 1, culture supernatant of *P. pastoris* transformed with blank pPIC9 vector (negative control); lanes 2–5, culture supernatant of *P. pastoris* transformed with the recombinant plasmids after 24, 48, 72, and 96 h of methanol induction. **(B)** Western blot identification of the recombinant proteins with the mouse anti–IBDV polyclonal antibody, the rabbit anti–chicken IgY and anti–His tag antibody. M, protein molecular size page ruler; lane 1, culture supernatant of *P. pastoris* transformed with blank pPIC9 vector (negative control); lane 2, culture supernatant of *P. pastoris* transformed with the recombinant plasmids at 96 h post induction. **(C)** Purification of the fused VP2. M, Page ruler pre–stained protein ladder; lane 1, culture supernatant after column chromatography; lane 2, purified VP2. **(D)** 3D structure of the recombinant proteins.

Initially, the VP2 and VP5 genes of IBDV and chicken IgY Fc gene were separately amplified by PCR, and then these genes were linked via overlapping PCR. The PCR products were cloned in the expression vector pPIC9 and verified by sequencing. Subsequently, the recombinant pPIC9-VP2, pPIC9-VP2–Fc, and pPIC9-VP2–VP5–Fc plasmids were transformed into *P. Pastoris*. Upon induction with methanol at different induction times, the novel expressed protein bands corresponding to 25.63, 49.35, and 67.09 kDa in the culture supernatants of the recombinant pPIC9-VP2, pPIC9-VP2–Fc, and pPIC9-VP2–VP5–Fc transformants were visualized through SDS-PAGE, respectively (Figure 1A). After 24 h of cultivation, the protein was detected in the supernatant apparently, and the maximum protein concentration (9.6, 8.3, and 13 mg/L) occurred at 72 h individually. Western blot analysis was performed with mouse anti-IBDV polyclonal antibody, anti-His tag antibody, and rabbit anti-chicken IgY antibody to determine the expression of the target proteins; we observed single reaction bands corresponding to the bands in SDS-PAGE, which indicates the expression of the recombinant VP2, VP2–Fc, and VP2–VP5–Fc proteins and their good reactogenicity to specific antibody (Figure 1B). Single-protein bands with molecular weights of 25.63, 49.35, and 67.09 kDa were detected in the result of SDS-PAGE after purification (Figure 1C). Moreover, at the start of the fusions, we predicted the 3D structures of the fusion proteins through homology modeling methods of SWISS-MODEL (Figure 1D).

### Immune effect comparison for serum antibody titers

**Figure 2:**
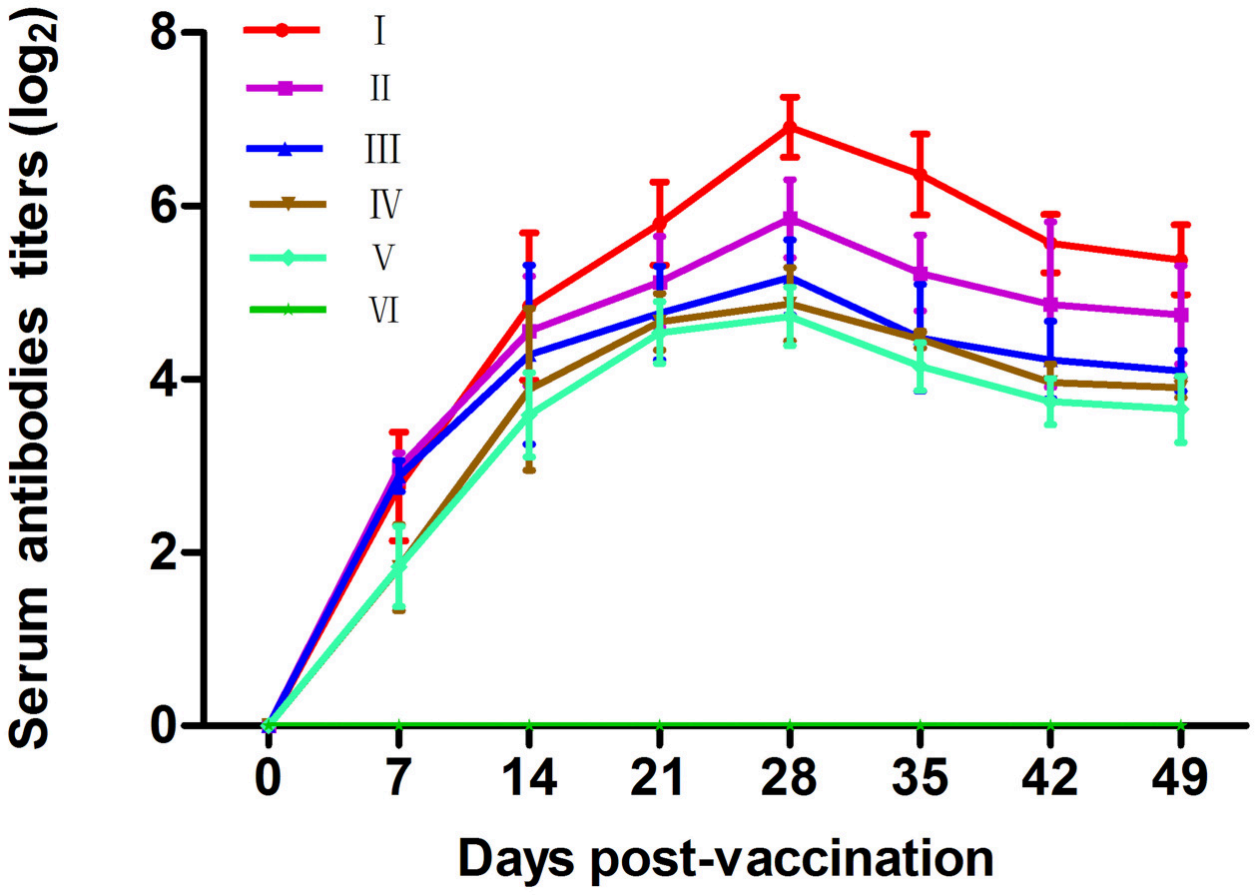
Changes of serum antibody titers in chickens. Chickens in six groups were vaccinated with TPPPS adjuvant VP2–VP5–Fc vaccine (I), pure VP2–VP5–Fc vaccine (II), pure VP2–Fc vaccine (III), pure VP2 vaccine (IV), commercial VP2 subunit vaccine (V), and PBS (VI) at 7 days old, respectively. Serum was collected at 0, 7, 14, 21, 28, 35, 42, and 49 dpv. Then the antibody titers were determined by indirect ELISA. All values shown are means ± *SD* from three independent experiments.

Antibody levels induced by vaccination are crucial to examine the effects of vaccines. The dynamic changes of serum antibody titers in each group are shown in Figure 2. The antibody levels in chickens of vaccine-inoculated groups were significantly higher than those in the chickens of the PBS group at 7–49 dpv (*P* < 0.05). Groups VP2–VP5–Fc+TPPPS and VP2–VP5–Fc had higher antibody titers at 14–49 dpv compared with other groups (*P* < 0.05). The antibody titers in group VP2–Fc were significantly higher than those in groups VP2 and commercial VP2 vaccine (*P* < 0.05), and the antibody titers in group VP2–VP5–Fc+TPPPS were significantly higher than those in group VP2–VP5–Fc (*P* < 0.05). In addition, the difference was insignificant between group VP2 and commercial VP2 subunit vaccine (*P* > 0.05). The results suggested that the recombinant protein VP2–VP5–Fc of IBDV had good immunogenicity, and the TPPPS effectively promoted the antibody titers induced by the recombinant subunit vaccine in chickens.

### Immune effect comparison for cytokine secretion

**Figure 3:**
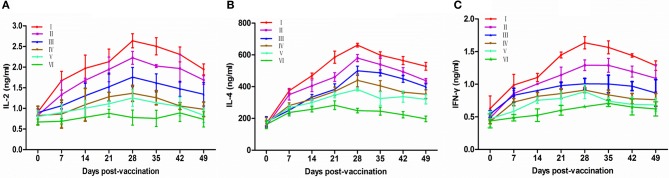
Changes of cytokines in chickens. Chickens in six groups were vaccinated with TPPPS adjuvant VP2–VP5–Fc vaccine (I), pure VP2–VP5–Fc vaccine (II), pure VP2–Fc vaccine (III), pure VP2 vaccine (IV), commercial subunit vaccine (V) and PBS (VI) at 7 days old, respectively. Serum was collected at 0, 7, 14, 21, 28, 35, 42, and 49 dpv. Then IL-2 **(A)**, IL-4 **(B)**, and IFN-γ **(C)** were detected by using the chicken IL-2, IL-4, and IFN-γ ELISA kits. All values shown are means ± *SD* from three independent experiments.

Cytokines are crucial for fighting off infections and are involved in immune responses (Lowry, 1993). For the secretion of cytokines, IL-2 and INF-γ mainly promote cell-mediated immune response, and IL-4 mainly promotes antibody production and mediates humoral immune responses. The concentrations of serum IL-2, IL-4, and IFN-γ in each group are shown in Figures 3A–C. In comparison with the PBS control group, the IL-2, IL-4, and IFN-γ secretions in all vaccine-inoculated groups showed an increase and reached the peak values at 28 dpv and that in PBS group at 21 dpv. IL-2, IL-4, and IFN-γ concentrations in groups VP2–VP5–Fc+TPPPS, VP2–VP5–Fc, and VP2–Fc were significantly higher than those in groups VP2 and commercial VP2 vaccine (*P* < 0.05). Apparently, the group inoculated with TPPPS as adjuvant showed higher concentrations of IL-2, IL-4, and IFN-γ relative to those of other groups at 14–49 dpv (*P* < 0.05). The results indicated that TPPPS and IgY Fc fragment significantly promoted the secretion of cytokines in chickens.

### Immune effect comparison for lymphocyte proliferation

**Figure 4:**
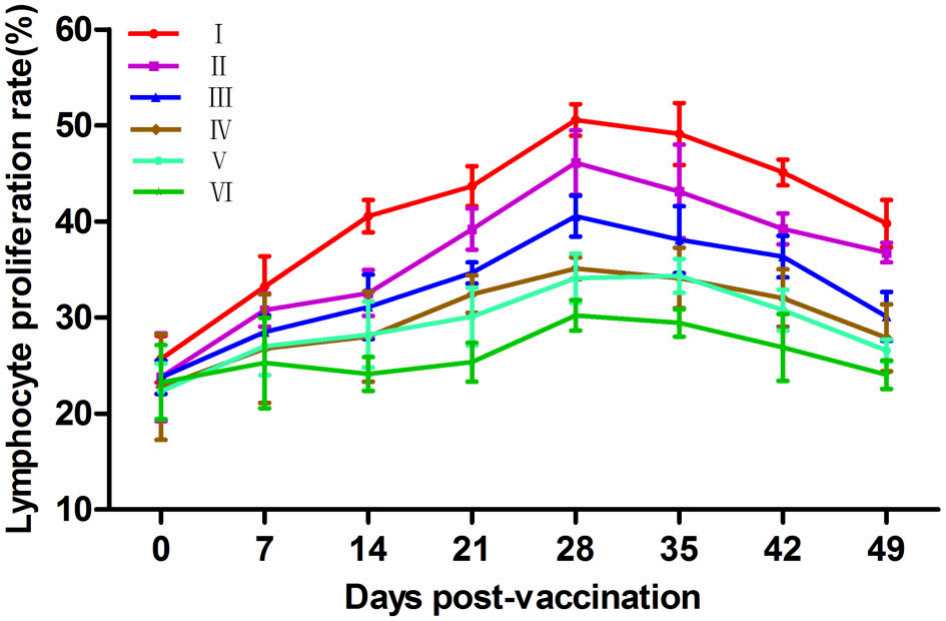
Changes of T lymphocyte proliferation ratio in chickens. Chickens in six groups were vaccinated with of TPPPS adjuvant VP2–VP5–Fc vaccine (I), pure VP2–VP5–Fc vaccine (II), pure VP2–Fc vaccine (III), pure VP2 vaccine (IV), commercial VP2 subunit vaccine (V), and PBS (VI) at 7 days old, respectively. Serum was collected at 0, 7, 14, 21, 28, 35, 42, and 49 dpv. The ratio of T lymphocyte proliferation in the same column was compared by Duncan's multiple–range tests. All values shown are means ± *SD* from three independent experiments.

Phytoagglutinins, such as ConA, can non-specifically stimulate mature T lymphocytes and lead to proliferation. The ratio of proliferation is commonly used to evaluate the cellular immunity (Asherson et al., 1973). The changes of lymphocyte proliferation rates are illustrated in Figure 4. At 14–49 dpv, the lymphocyte transformation rates (LTRs) in groups VP2–VP5–Fc+TPPPS, VP2–VP5–Fc, and VP2–Fc were significantly higher than those in groups VP2, commercial VP2 vaccine, and PBS (*P* < 0.05). Notably, the group VP2–VP5–Fc+TPPPS showed significantly higher LTRs than group VP2–VP5–Fc at 14–49 dpv (*P* < 0.05). Furthermore, the LTRs in group VP2–VP5–Fc were significantly higher than those in group VP2–Fc (*P* < 0.05). These results suggested that TPPPS and linked Fc significantly promoted humoral and cellular immune responses induced by the VP2–VP5 antigen.

### Immune effect comparison for lymphocyte subsets ratio

CD4^+^ and CD8^+^ percentages directly reflect immune function in animals (Torti et al., 2012). The changes in CD4^+^ and CD8^+^ T lymphocyte counts in peripheral blood are illustrated in Tables 2, 3, respectively. The levels of CD4^+^ and CD8^+^ in groups VP2–VP5–Fc+TPPPS, VP2–VP5–Fc, VP2–Fc, VP2, and commercial VP2 vaccine were significantly higher than those in group PBS at 14–42 dpv (*P* < 0.05). Furthermore, the percentages of CD4^+^ in group VP2–VP5–Fc+TPPPS inoculated with TPPPS were significantly higher than those in other groups (*P* < 0.05). The percentages of CD4^+^ in group VP2–Fc were significantly higher than those in group VP2 (*P* < 0.05); the percentages of CD4^+^ in group VP2 were higher than those in group commercial VP2 vaccine, and the difference was insignificant (*P* > 0.05). Similarly, the percentages of CD8^+^ T lymphocytes did not show a difference compared with CD4^+^ T lymphocytes. The results revealed that TPPPS and linked Fc effectively improved the percentages of CD4^+^ and CD8^+^ T lymphocytes in the in peripheral blood of chickens.

**Table 2:** Changes of CD4^+^ T lymphocyte counts in the peripheral blood.

**Group^A^**	**Days post–vaccination^B^**							
	**0**	**7**	**14**	**21**	**28**	**35**	**42**	**49**
I	19.30 ± 0.04^ab^	22.65 ± 0.80^ab^	26.39 ± 0.65^a^	29.62 ± 0.21^a^	31.44 ± 0.50^a^	37.04 ± 0.49^a^	32.41 ± 1.02^a^	31.40 ± 0.99^a^
II	19.91 ± 0.58^a^	23.31 ± 0.75^a^	23.63 ± 0.85^b^	25.24 ± 0.70^b^	26.38 ± 0.58^b^	31.09 ± 1.27^b^	29.95 ± 0.84^b^	28.81 ± 1.23^b^
III	19.25 ± 0.88^ab^	21.45 ± 1.09^ab^	21.83 ± 0.53^bc^	22.96 ± 0.83^c^	23.99 ± 0.37^c^	25.59 ± 0.54^c^	24.64 ± 0.64^c^	24.26 ± 0.67^c^
IV	18.39 ± 0.79^b^	20.74 ± 0.43^bc^	22.66 ± 0.88^cd^	21.25 ± 0.11^de^	21.74 ± 1.10^d^	23.09 ± 0.84^d^	22.40 ± 0.35^d^	22.24 ± 0.62^d^
V	18.27 ± 0.23^b^	19.01 ± 1.77^c^	20.90 ± 1.10^d^	22.03 ± 0.17^d^	22.00 ± 0.68^d^	22.95 ± 0.94^d^	21.45 ± 0.78^d^	20.90 ± 0.31^de^
VI	19.59 ± 0.43^a^	20.78 ± 1.96^bc^	20.67 ± 1.03^d^	20.79 ± 0.37^e^	20.47 ± 0.31^e^	20.75 ± 0.80^e^	20.94 ± 0.17^e^	20.68 ± 0.41^e^

A *Group names represent that chickens in these groups were vaccinated with TPPPS adjuvant VP2–VP5–Fc vaccine (I), pure VP2–VP5–Fc vaccine (II), pure VP2–Fc vaccine (III), pure VP2 vaccine (IV), commercial VP2 subunit vaccine (V), and PBS (VI) at 7 days old, respectively*.

B *CD4^+^ T lymphocyte counts in the same column were compared by Duncan's multiple–range tests. Different lowercase letter superscripts indicate significant differences (P < 0.05). Data are expressed as mean percentage ± SD*.

**Table 3:** Changes of CD8^+^ T lymphocyte counts in the peripheral blood.

**Group^A^**	**Days post–vaccination^B^**							
	**0**	**7**	**14**	**21**	**28**	**35**	**42**	**49**
I	13.01 ± 0.53^a^	14.21 ± 0.35^a^	16.12 ± 0.62^a^	18.73 ± 0.83^a^	20.50 ± 0.78^a^	20.26 ± 0.20^a^	19.52 ± 0.21^a^	18.88 ± 0.58^a^
II	11.83 ± 0.50^c^	13.87 ± 0.50^b^	15.05 ± 0.50^ab^	16.53 ± 0.30^b^	18.20 ± 0.46^b^	18.57 ± 0.96^b^	18.21 ± 0.28^b^	16.80 ± 0.43^b^
III	12.21 ± 0.49^abc^	12.67 ± 0.56^bc^	14.24 ± 0.47^bc^	15.21 ± 0.55^c^	17.51 ± 1.01^b^	17.44 ± 0.56^b^	16.94 ± 0.48^c^	15.55 ± 0.29^c^
IV	12.78 ± 0.41^ab^	12.95 ± 0.33^b^	13.05 ± 0.61^cd^	14.43 ± 0.74^cd^	15.46 ± 0.48^c^	16.15 ± 0.31^c^	15.43 ± 0.30^d^	13.70 ± 0.16^d^
V	12.90 ± 0.12^a^	12.76 ± 0.30^a^	12.32 ± 1.30^de^	13.55 ± 0.78^de^	15.43 ± 0.89^c^	14.75 ± 0.87^d^	14.36 ± 0.50^e^	13.24 ± 0.50^d^
VI	12.06 ± 0.32^bc^	12.00 ± 0.13^c^	11.34 ± 0.54^e^	12.52 ± 0.66^f^	13.15 ± 1.15^d^	13.59 ± 0.99^d^	13.13 ± 0.23^f^	12.19 ± 0.73^e^

A *Group names represent that chickens in these groups were vaccinated with of TPPPS adjuvant VP2–VP5–Fc vaccine (I), pure VP2–VP5–Fc vaccine (II), pure VP2–Fc vaccine (III), pure VP2 vaccine (IV), commercial VP2 subunit vaccine (V), and PBS (VI) at 7 days old, respectively*.

B *CD8^+^ T lymphocyte counts in the same column were compared by Duncan's multiple–range tests. Different lowercase letter superscripts indicate significant differences (P < 0.05). Data are expressed as mean percentage ± SD*.

### Protective effects of IBDV subunit vaccines

**Figure 5:**
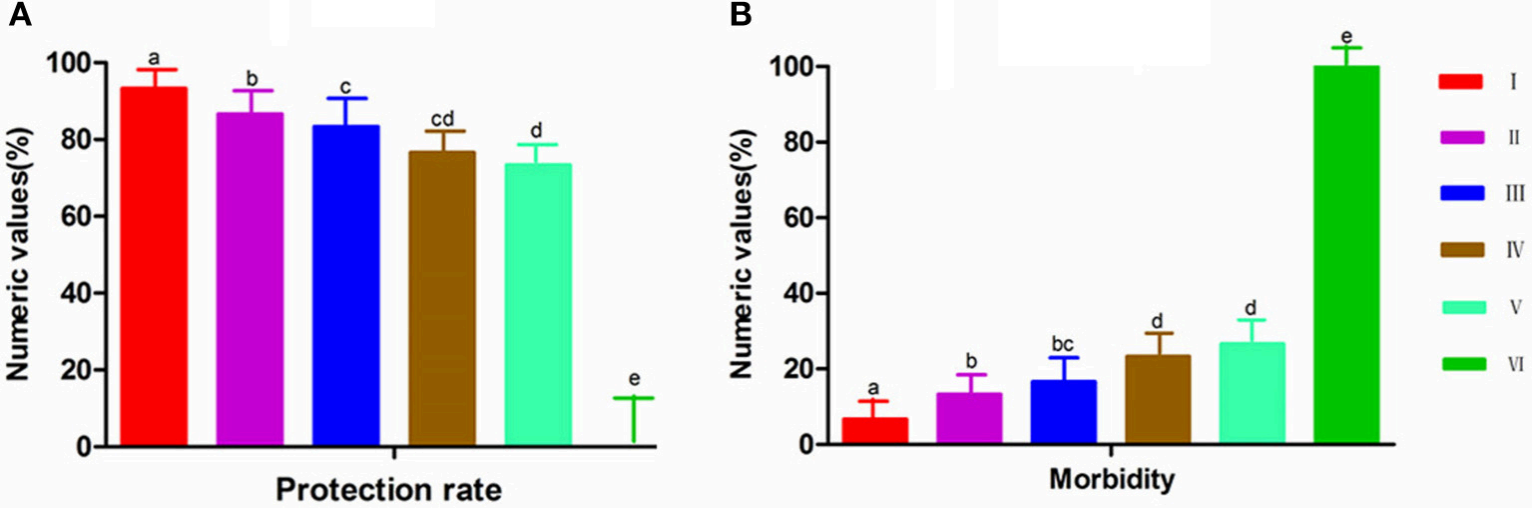
Protection **(A)** and morbility **(B)** rates of chickens challenged with IBDV in different groups. Chickens in six groups were vaccinated with TPPPS adjuvant VP2–VP5–Fc vaccine (I), pure VP2–VP5–Fc vaccine (II), pure VP2–Fc vaccine (III), pure VP2 vaccine (IV), commercial VP2 subunit vaccine (V), and PBS (VI) at 7 days old, respectively. Thirty chickens from each group were challenged with 2,000 ELD_50_ vvIBDV GX8/99 strain. Clinical symptoms and survival status were observed and recorded for 7 successive days after challenge. Different lowercase letters above the columns indicate significant differences between the different groups (*P* ≤ 0.05).

**Figure 6:**
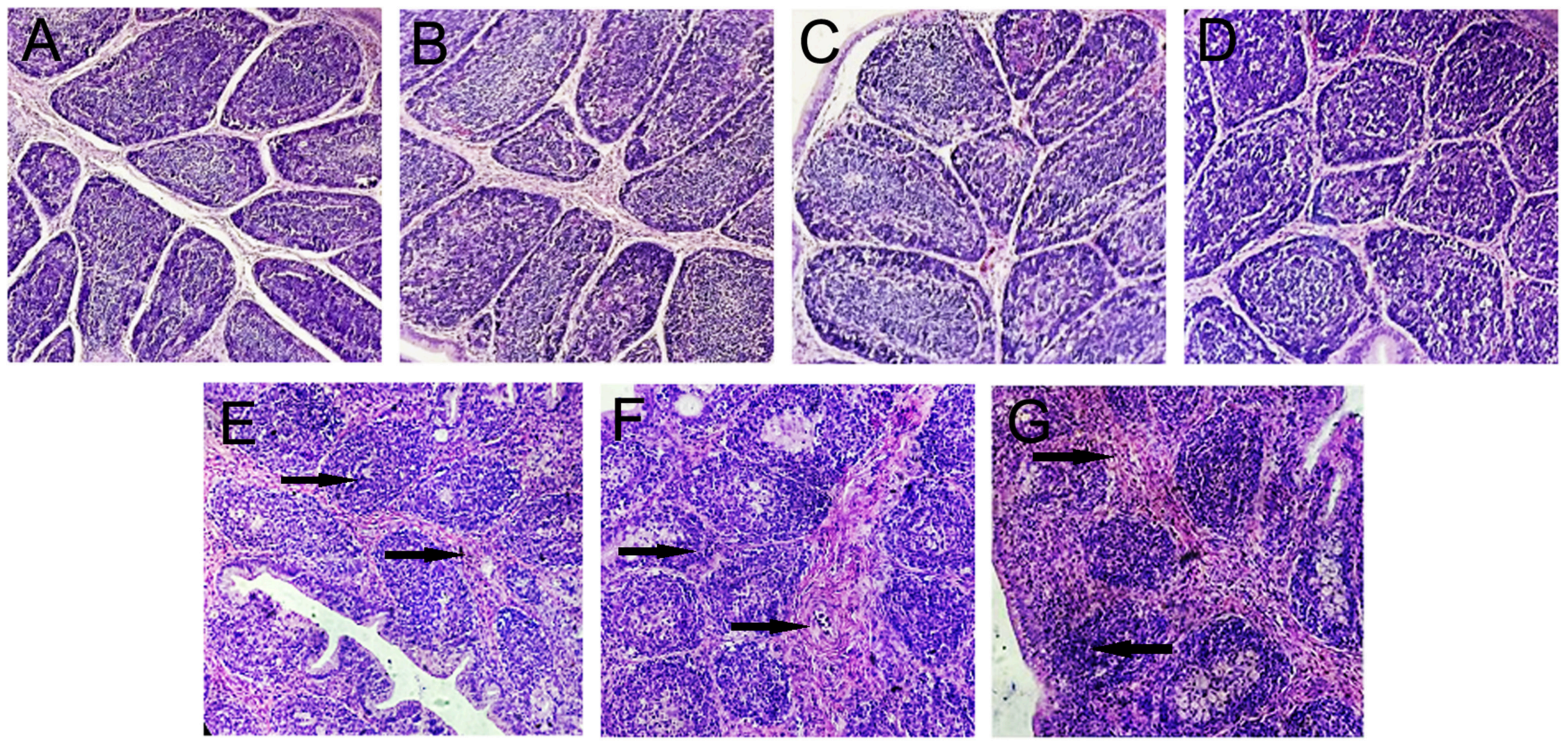
Bursal histopathological characteristics. Chickens were non-infected and treated with PBS as normal control **(A)**. Chickens in six groups were vaccinated with TPPPS adjuvant VP2–VP5–Fc vaccine **(B)**, pure VP2–VP5–Fc vaccine **(C)**, pure VP2–Fc vaccine **(D)**, pure VP2 vaccine **(E)**, commercial VP2 subunit vaccine **(F)**, and PBS **(G)** at 7 days old, respectively. Thirty chickens from each group were challenged intranasally with 2,000 ELD_50_ virulent IBDV. The arrows indicate necrosis of the follicles, lymphoid depletion and fibroplasia of bursal.

Thirty inoculated chickens from each group were challenged with 2,000 ELD_50_ of the virulent IBDV to evaluate the protective effects of the subunit vaccines against IBDV infection. In the monitoring period, clinical symptoms and survival status of the chickens were recorded daily, and the protective rates and morbidity of different groups are illustrated in Figures 5A,B. After 7 days of observation, 100% of chickens in the PBS group showed symptoms. By contrast, the morbidity of chickens vaccinated with subunit vaccines was significantly lower than those in group PBS. Moreover, the protection rate in group VP2–VP5–Fc+TPPPS was significantly higher than those in group VP2–VP5–Fc (*P* < 0.05), the protection rate in group VP2–Fc was significantly higher than those in group VP2 (*P* < 0.05), and the difference between groups VP2 and commercial VP2 subunit vaccine was insignificant (*P* > 0.05). Furthermore, bursal lesion scores based on bursal histopathological characteristics are presented in Figure 6. Chickens in group A (unchallenged) showed a normal morphology, and those in other groups (B, C, D) showed almost normal morphology. By contrast, chickens in groups (E and F) presented a little lymphoid follicular dysplasia, and chickens in group G showed lymphoid follicular dysplasia, reduced lymphocytes, and loose lymphocyte arrangement in medulla area. These data demonstrated that the yeast-expressed VP2–VP5–Fc and VP2–Fc effectively protect chickens against IBDV infection, whereas the VP2–VP5–Fc subunit vaccine combined with TPPPS can reach the optimal protection.

## Discussion

IBDV still poses a huge threat to the poultry industry. The immunogenicity of current IBDV subunit vaccines was limited; therefore, how to improve the immunogenicity of IBDV subunit vaccine is crucial for the prevention and control of this disease. In the present study, we fused VP2 and VP5 protective antigen genes and linked them to the chicken IgY Fc gene, thus constructing the eukaryotic expression system of VP2–VP5–Fc. Meanwhile, TPPPS was added to the expressed products to verify its immune enhancement effects on the subunit vaccine. The immunogenicity of VP2–VP5–Fc recombinant protein expressed in eukaryotic system was significantly higher than the commercial VP2 vaccine, and the use of TPPPS can further improve the immune response.

VP2 protein, a target protein used to construct recombinant vaccines, has been expressed in a variety of systems and has been shown to exhibit immunogenicity and induce an immune response (Müller et al., 2003). Moreover, VP5 protein also possesses certain immunogenicity (Zhang et al., 2007). Therefore, linking these two gene fragments together is advantageous considering the relevant immunogenicity of them. Judging from this study, the recombinant protein linked with VP5 protein showed a better immunogenicity compared with the VP2–Fc protein. These results indicated that recombinant VP2–VP5–Fc subunit vaccine was able to exert better immunogenicity, thereby inducing the corresponding immune response to protect chickens from IBDV challenge.

Antibodies administered concurrently with an antigen can modulate the immune response of antigens, which is called antibody-mediated feedback (Brady, 2005), and the Fc region mediates potent immune effector functions by engaging FcRs and serum complement proteins (Sang, 2013), hence, providing new opportunities for augmenting the immunogenicity of antigens. In this study, we synthetically linked VP2 and recombinant VP2-VP5 fragments to IgY Fc via the flexible hinge region to guarantee the independent space structure of the fused sections. After verification, recombinant subunit vaccine linked with IgY Fc segment showed distinct advantages in terms of immune potency, and the immunological function of Fc has been fully affirmed. These results imply that the linked chicken IgY Fc improved vaccine immunogenicity of the recombinant protein and enhanced the immune responses in chickens.

*P. pastoris* expression system was used to express the recombinant VP2, VP2–Fc, and VP2–VP5–Fc proteins. This eukaryotic expression presented several advantages, such as easy cultivation, high yield, precise post-translational modifications, and minimal interference of native proteins (Cereghino and Cregg, 2000). Furthermore, this secretory expression system facilitates subsequent purification (Cereghino and Cregg, 2000; Luo et al., 2009), thereby avoiding damage to the content and activity of the recombinant fusion proteins. Based on our results, these foreign proteins were monitored in the medium with high levels. In comparison, the amount of native proteins of *P. pastoris* secreted was in low quantity, which greatly improved the purification efficiency of the recombinant fusion proteins. Thus, the *P. pastoris* expression system is suitable for expressing the recombinant fusion proteins.

TPPPS has been demonstrated that it can enhance the stimulating abilities of vaccines to induce better humoral and cellular immunities (Zhao et al., 2013; Guo et al., 2014). Similarly, judging from our results, TPPPS used as adjuvant exhibited a big enhancement for the immune response of the recombinant subunit. Thus, TPPPS can serve as a potential adjuvant for novel IBD subunit vaccine.

## Conclusion

Altogether, four ways were used to improve the immune effect of IBDV subunit vaccine in this study. We found that the immune effects of recombinant IBD subunit vaccine have been effectively improved through these four approaches. The fused VP2–VP5–Fc subunit can significantly enhance the specific immune response and reduce morbidity and mortality compared with the traditional subunit vaccine. Moreover, TPPPS as the adjuvant showed good immune enhancement on the fused subunit vaccine. In summary, our results provide a novel prospect for improving the immune efficacy of the IBD subunit vaccine.

## Author contributions

RZ, KW, HW, and SS designed research; HW, SS, SW, HZ, LM, LH, and HH performed research; HW, SS, RZ, and KW analyzed data; HW, SS, RZ, and KW wrote the paper.

### Conflict of interest statement

The authors declare that the research was conducted in the absence of any commercial or financial relationships that could be construed as a potential conflict of interest.
